# Ki-67 as a Marker to Differentiate Burkitt Lymphoma and Diffuse Large B-cell Lymphoma: A Literature Review

**DOI:** 10.7759/cureus.72190

**Published:** 2024-10-23

**Authors:** Nicyela J Harlendea, Kent Harlendo

**Affiliations:** 1 Pathology, Tarumanagara University, Jakarta, IDN; 2 Clinical Pathology, Sebelas Maret University, Solo, IDN

**Keywords:** burkitt lymphoma, diagnostic marker, diffuse large b-cell lymphoma, ki-67, prognostic marker

## Abstract

Burkitt lymphoma (BL) is a form of non-Hodgkin’s lymphoma (NHL) that is characterized by high aggressiveness and arises from the germinal center of B cells. The prevalence of BL in adulthood is less than 5%. However, it encompasses 40% of all childhood NHL. Diffuse large B-cell lymphoma (DLBCL) is the most common lymphoma. It accounts for approximately 25% of all NHL cases worldwide. The differentiation between BL and DLBCL is more clear in theory than in daily practice. However, it is important because it implies different treatments. Compared to the other indolent small cell lymphomas, DLBCLs and BLs show higher Ki-67 index values. The Ki-67 levels in DLBCL typically range from 40% to 90%, while BL has a high Ki-67 positivity, nearing 100%. The aim of this article is to explore and review the function of Ki-67 as a differential marker for BL and DLBCL. An all-language literature search was conducted on MEDLINE, Cochrane, Embase, and Google Scholar until March 2024. The following search strings and Medical Subject Heading (MeSH) terms were used: "Ki-67," “Burkitt lymphoma," and “diffuse large B-cell lymphoma." We comprehensively reviewed the literature on BL, DLBCL, and the Ki-67 marker.

## Introduction and background

Distinguishing between Burkitt lymphoma (BL) and diffuse large B-cell lymphoma (DLBCL) is more challenging in practice than in theory [1]. BL has the characteristics of medium-sized lymphoma cells, displaying a uniform distribution, and has small nucleoli. The blue-round tumor cells have tangible-bodied macrophages, clear cytoplasm, and scattered apoptotic bodies resulting in a distinctive "starry-sky" look. BL has a high proliferative index [2]. DLBCL encompasses a diverse collection of mature B-cell neoplasms ranging from moderate to high grade. Their cells are typically larger and have more size variability, cytoplasm with vesicular chromatin, and prominent nucleoli. Both lymphomas express mature B-cell markers [3]. Classic BL has an almost 100% proliferation index and was previously known to test negative for BCL2, but according to the current WHO classification, up to 20% of cases have a weak expression of BCL2 [4]. Conversely, DLBCL typically has a proliferation index of less than 90% and often tests positive for BCL2 [3]. The Ki-67 labeling index, a commonly used marker of proliferation in oncology, is typically determined by counting cells that are positively stained out of the total number of cells [5]. While DLBCL frequently has a Ki-67 index ranging from 40% to 90%, BL has a proliferation index of nearly 100% [4,6]. For that reason, Ki-67 may be a good differential marker for BL and DLBCL [5].

Ki-67 as a diagnostic marker and differential marker has been well-documented and extensively studied. Despite this, significant gaps in the literature exist. This literature review aims to explore and summarize the current literature on Ki-67 as a differential marker for BL and DLBCL. We conducted an all-language literature search on MEDLINE, Cochrane, Embase, and Google Scholar until March 2024. The following search strings and Medical Subject Heading (MeSH) terms were used: "Ki-67," “Burkitt lymphoma," and “diffuse large B-cell lymphoma." We comprehensively reviewed the literature on BL, DLBCL, and the Ki-67 marker.

## Review

BL

BL is a very aggressive form of B-cell non-Hodgkin's lymphoma (NHL) for which Denis Burkitt initially identified in 1958. This lymphoma originates from the germinal center of B cells. The development of BL depends on the expression of the MYC gene, which encodes the transcription factor c-myc protein, which is located on chromosome 8q24 and regulates cell proliferation, differentiation, and apoptosis [7-9]. BL is marked by highly elevated levels of c-myc, which can arise through several mechanisms, with the most prevalent being the translocation of the long arm of chromosome 8 (which contains the MYC gene) and the immunoglobulin (Ig) heavy chain gene on chromosome 14. The overexpression of c-myc results in accelerated B-cell proliferation, which in turn leads to a shortened doubling time of BL tumor cells [8].

Epidemiology

While BL affects less than 5% of adults, it accounts for 40% of all NHL cases in children [10]. BL can be divided into three clinical groups: endemic, sporadic, and immunodeficiency-related. The endemic form of BL (eBL) is the most prevalent variation [11]. eBL is a pediatric cancer, with the highest occurrence observed between the ages of six and eight. It is prevalent in areas with perpetual malaria transmission, mainly in Sub-Saharan Africa and Papua New Guinea [12]. The endemic form is associated with malaria and Epstein-Barr Virus (EBV) [11]. EBV is linked to over 95% of endemic BL. Conversely, the percentage of sporadic BLs that are positive for EBV is just 5%-15%, while 40% of immunodeficiency-associated BLs are positive for EBV [13]. The exact mechanisms remain unclear [14]. In contrast to eBL, sporadic BL cases are found in developed countries and accounts for 1%-2% of lymphomas in adults and 30%-40% of lymphomas in children [15]. The immunodeficiency-related BL accounts for approximately 20% of BL cases in the United States. Immunodeficiency-associated BL does not just occur to HIV patients but also affects individuals with congenital immunodeficiency and recipients of allografts. Its prevalence has not changed significantly in HIV patients with the advent of highly active antiretroviral therapy (HAART) [16,17].

Clinical Features

BL often presents as a rapidly growing tumor and is characterized by a very short doubling time of between 24 and 48 hours and rapid spread to extranodal sites, including the central nervous system (CNS) and bone marrow. Nearly 70% of newly diagnosed patients had stage III or advanced stage IV disease at the time of diagnosis. BL at the time of diagnosis may be associated with spontaneous tumor lysis syndrome (TLS), which requires early transfer to the intensive care unit (ICU) [18]. The abdomen is the primary site for sporadic BL, even though it can also affect the head and neck. BL symptoms (i.e., fever, night sweats, weight loss) are more likely to be seen in adult patients. At the time of initial diagnosis, 30% and 15% of patients show bone marrow and CNS involvement [2]. The most common site of onset of the endemic form is the jaw, which is present in 50% of cases and occurs more frequently in young patients (the peak age of incidence is three to seven years). Typical presentations of endemic BL frequently feature the presence of enlarging jaw lesions, periorbital lesions, or involvement of the genitourinary system. Jaw involvement is seen mainly in children. Malnutrition often occurs at the time of disease presentation [2]. Patients with immunodeficiency-associated BL often present with signs and symptoms associated with immunodeficiency and lymph node involvement, with an increased risk of CNS spread [16].

Morphological and Histological Features

**Figure 1:**
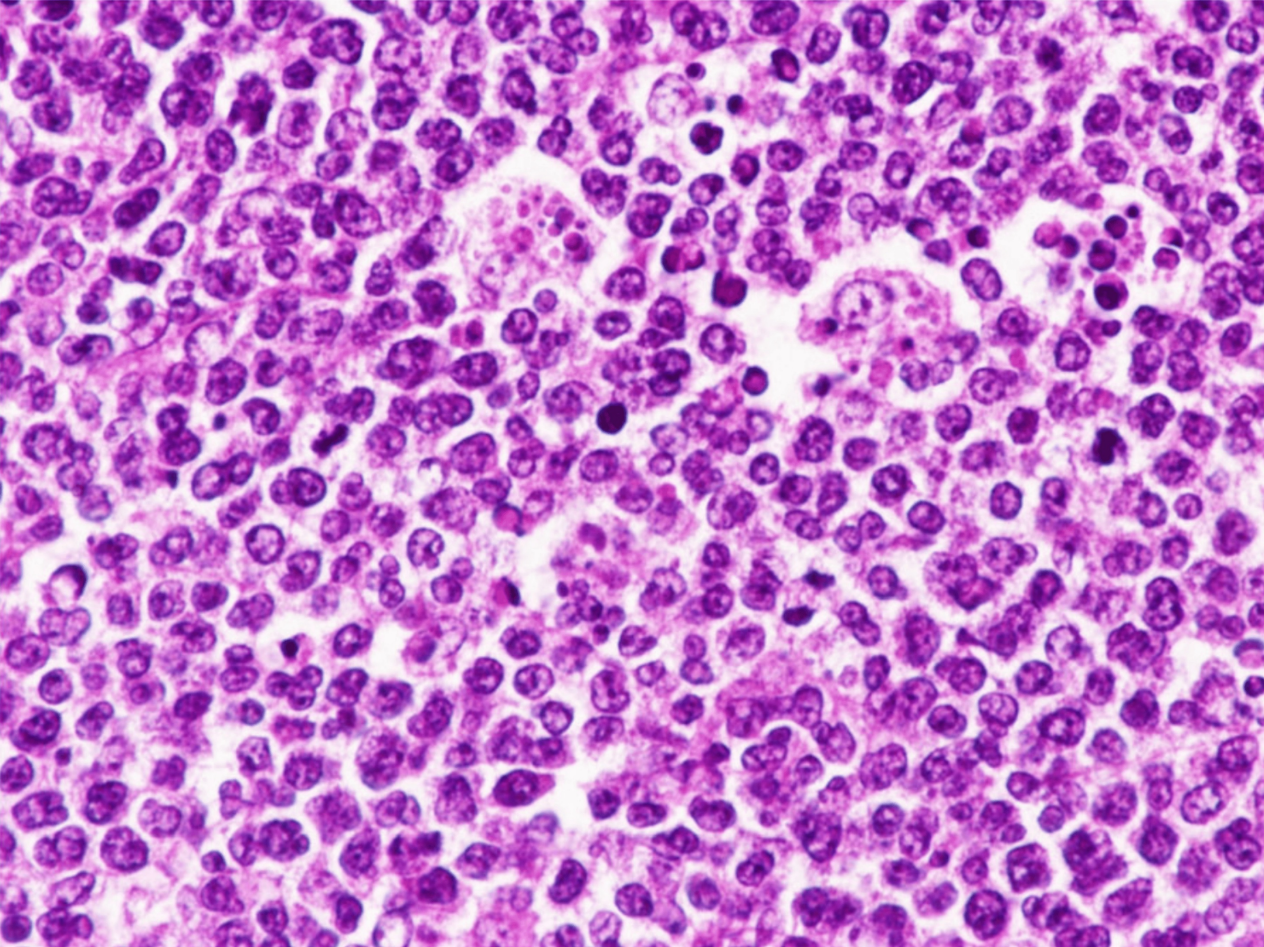
A notable infiltration of cells with strong basophilia, medium-sized lymphocytes, and some prominent nucleoli which are distinctive features of the starry sky pattern associated with Burkitt lymphoma (magnification x400) Image reproduced with permission. Reprinted from [20]

BL is a highly aggressive form of B-cell lymphoma characterized by a homogeneous population of intermediate-sized mature lymphocytes. The cells contain round nuclei characterized by lacy chromatin and may have one or more small nucleoli. BL is histologically characterized by the complete thinning of the lymph node architecture by sheets of lymphocytes. Tumor cells are usually medium-sized and nonpleomorphic and contain basophilic cytoplasm, prominent vacuoles, and round nuclei. A large number of large, irregularly shaped macrophages, which have consumed apoptotic tumor cells, are scattered among the lymphocytes, giving them the classic “starry sky” feature (Figure 1) [19,20]. 

Immunohistochemical Findings

**Figure 2:**
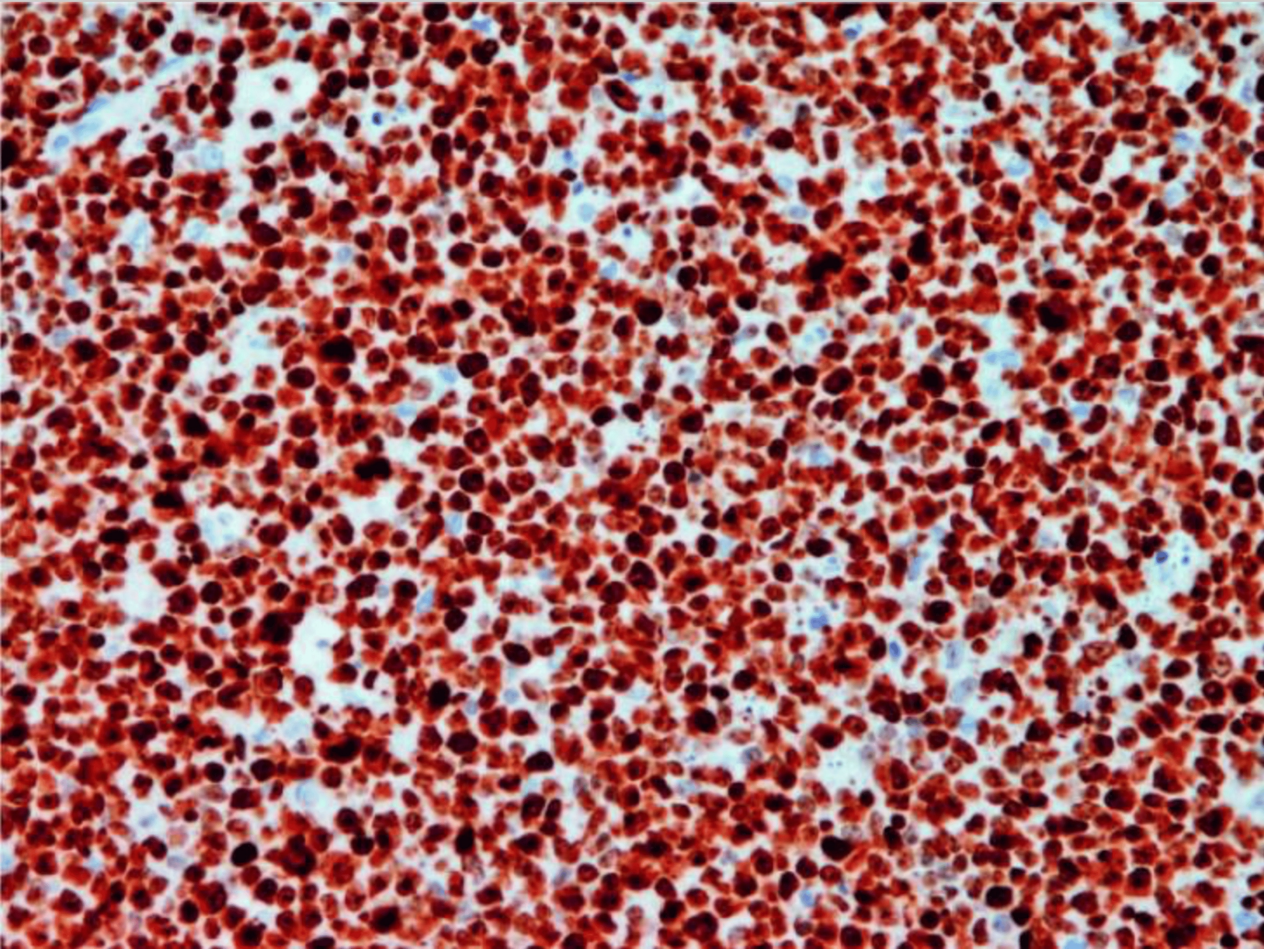
The immunohistochemistry of Burkitt lymphoma exhibits nearly 100% nuclear positive for Ki-67, indicating a high level of cell proliferation (magnification x200) Image reproduced with permission. Reprinted from [23]

Immunohistochemistry (IHC) is being extensively utilized as an essential tool in pathology to determine a diagnosis, as well as for BL. IHC is a method that utilizes the binding of antigen and antibody to identify specific antigen present in cells and tissues [2,21]. The tumor cells in BL possess a significant level of surface IgM expression that can vary from moderate to strong. The cells exhibited positive expression of B-cell markers, namely, CD19, CD20, CD22, CD79a, and PAX5. Their germinal center markers CD10 and Bcl-6 are positive, but BCL-2 is negative. The neoplastic cells lack the expression of T cell markers and do not exhibit the presence of terminal deoxynucleotidyl transferase (TdT) or immature CD34 signals. They are usually also negative for CD5, CD23, and CD138. High Ki-67 positivity, close to 100%, reflects rapid cell turnover and is a very helpful diagnostic clue (Figure 2) [2,22,23].

Management

High-dose multiagent chemotherapy has consistently been the primary approach for treating adult BL [24]. Over the past three decades, Western countries have developed a treatment approach that relies on two primary chemotherapy protocols. These protocols are based on the Lymphomes malins B (LMB) and Berlin-Frankfurt-Münster (BFM) regimens. These two treatment plans are highly comparable worldwide, as they both depend on a brief but intense combination of chemotherapy medications that are not resistant to each other, along with intrathecal prophylaxis [25]. The LMB therapy plan involves the administration of prednisone, vincristine, cyclophosphamide, doxorubicin, methotrexate, cytarabine, and etoposide [26]. The BFM protocol consists of dexamethasone, cyclophosphamide, vincristine, ifosfamide, cytarabine, etoposide, doxorubicin, and methotrexate. When rituximab is administered together with it, the regimen is referred as BFM-NHL-90. In 1996, Margrath and peers from the National Cancer Institute (NCI) reported their findings on the CODOX-M/IVAC regimens [19]. The CODOX-M/IVAC protocol usually consists of cyclophosphamide, vincristine, doxorubicin, and methotrexate (CODOX-M section) and etoposide, ifosfamide, and cytarabine (IVAC section) [27]. A study by Oosten et al. shows that these treatment protocols come up with almost comparable outcomes in terms of effectiveness and safety but vary in terms of treatment length and costs [28]. Combining rituximab with aggressive chemotherapeutic strategies results in overall survival rates of approximately 75% to 85% [19]. At present, the used protocols have demonstrated overlapping efficacy and toxicity in practical applications. The higher toxicity rate corresponds to the advancing age, leading to a reduction in the expected therapeutic dosage [24]. Therefore, older adults have a more unfavorable prognosis [29]. 

DLBCL

DLBCL is the most common type of NHL, representing around 30%-40% of cases worldwide [30]. DLBCL is defined by the widespread proliferation of large, mature B cells. These cells are generally larger than or equal to twice the average size of macrophages or lymphocytes. The BCL-2 protein plays a crucial role in the development of NHL [31]. The translocation t(14;18), where BCL-2 is located on chromosome 18 and the heavy Ig chain is on chromosome 14, leads to overexpression of the BCL-2 protein, which occurs in approximately 35% of DLBCL cases [32] While the BCL-6 gene has genetic alterations in approximately 20% to 40% of individuals [33].

Epidemiology

The average yearly incidence of NHL in the United States is roughly 7 cases per 100,000 individuals. DLBCL constitutes approximately 25% of all instances of NHL globally. DLBCL is the most prevalent type of NHL, with follicular lymphoma being the second most common. The disease exhibits a greater prevalence among Caucasians, followed by African-Americans and Asians, with the highest incidence reported in males and a median age of 64 years. The overall occurrence rises significantly with advancing age. The global incidence of NHL has increased by 35% over the past two decades. The estimated incidence of NHL is expected to be 5 cases per 100,000 individuals, with a mortality rate of 2.5 cases per 100,000 individuals [34].

Clinical Features

DLBCL commonly manifests as enlarged lymph nodes or a quickly expanding tumor, followed with B symptoms such as fever, night sweats, and weight loss. Approximately 30% of patients may encounter B symptoms. Bone marrow involvement can be observed in about 50% of less severe diseases. Roughly half of the patients exhibit extranodal involvement, which can present in various sites such as the skin, bones, spinal cord, and testicles [33].

Histological and Morphological Features

**Figure 3:**
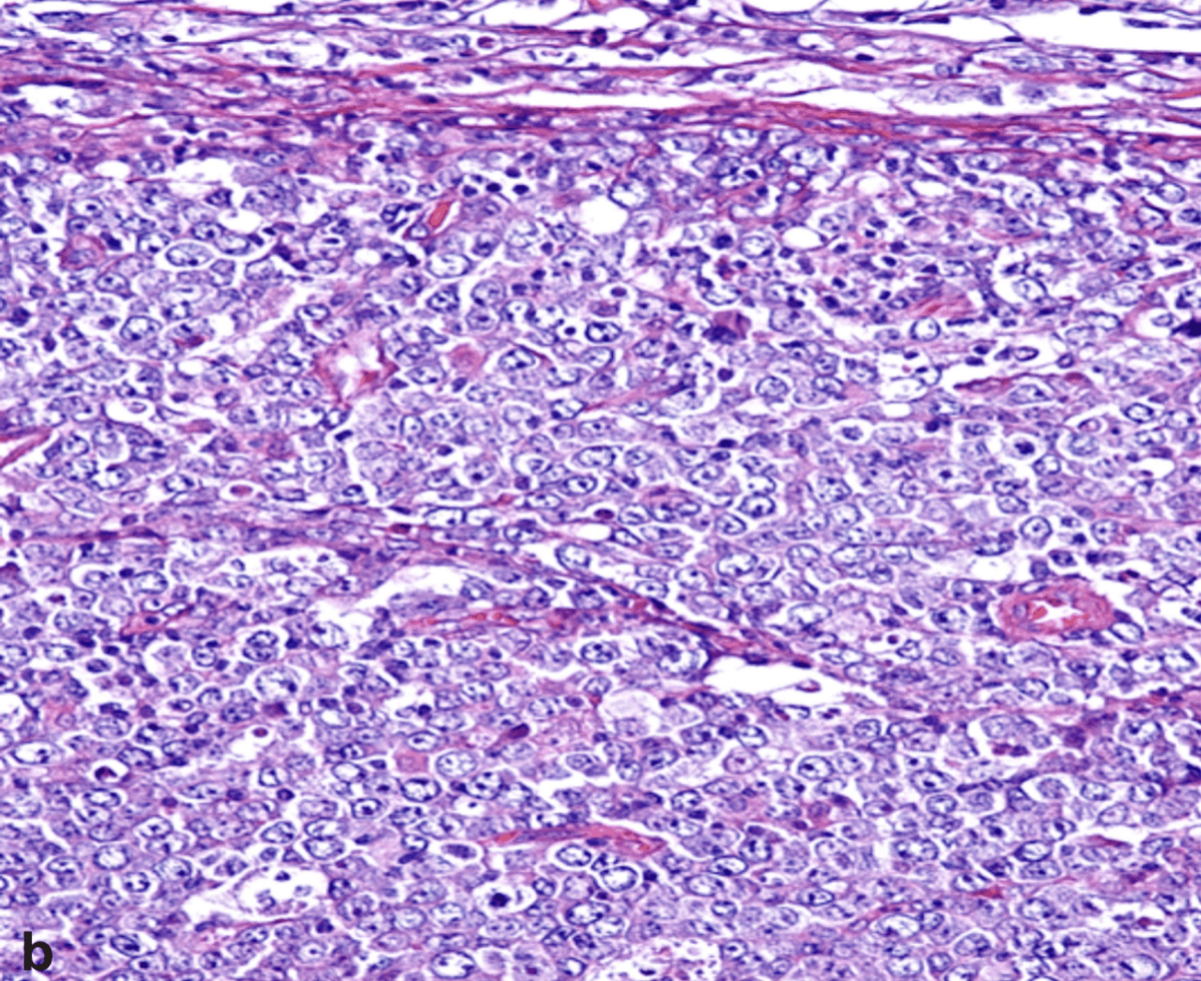
The morphological image of DLBCL demonstrates diffuse lymphoid proliferation that features the presence of large cells with round nucleus and prominent nucleoli (magnification x400). DLBCL: Diffuse large B-cell lymphoma Image reproduced with permission. Reprinted from [35]

The term diffuse large B-cell lymphoma itself describes the morphology of the disease. The lymphoma cells exhibit large sizes and are organized in a diffuse manner (Figure 3), causing complete or partial destruction of the normal structure of the lymph nodes or surrounding tissues. The typical morphology of lymph nodes is altered and replaced by tiers of atypical lymphoid cells with enlarged nuclei, basophilic cytoplasm, and a high rate of cell proliferation [33]. Slight fibrosis can divide clusters of lymphoma cells, or the tumor may be associated with sclerosis. Geographic necrosis may appear in certain areas [30].

Immunohistochemical Findings

**Figure 4:**
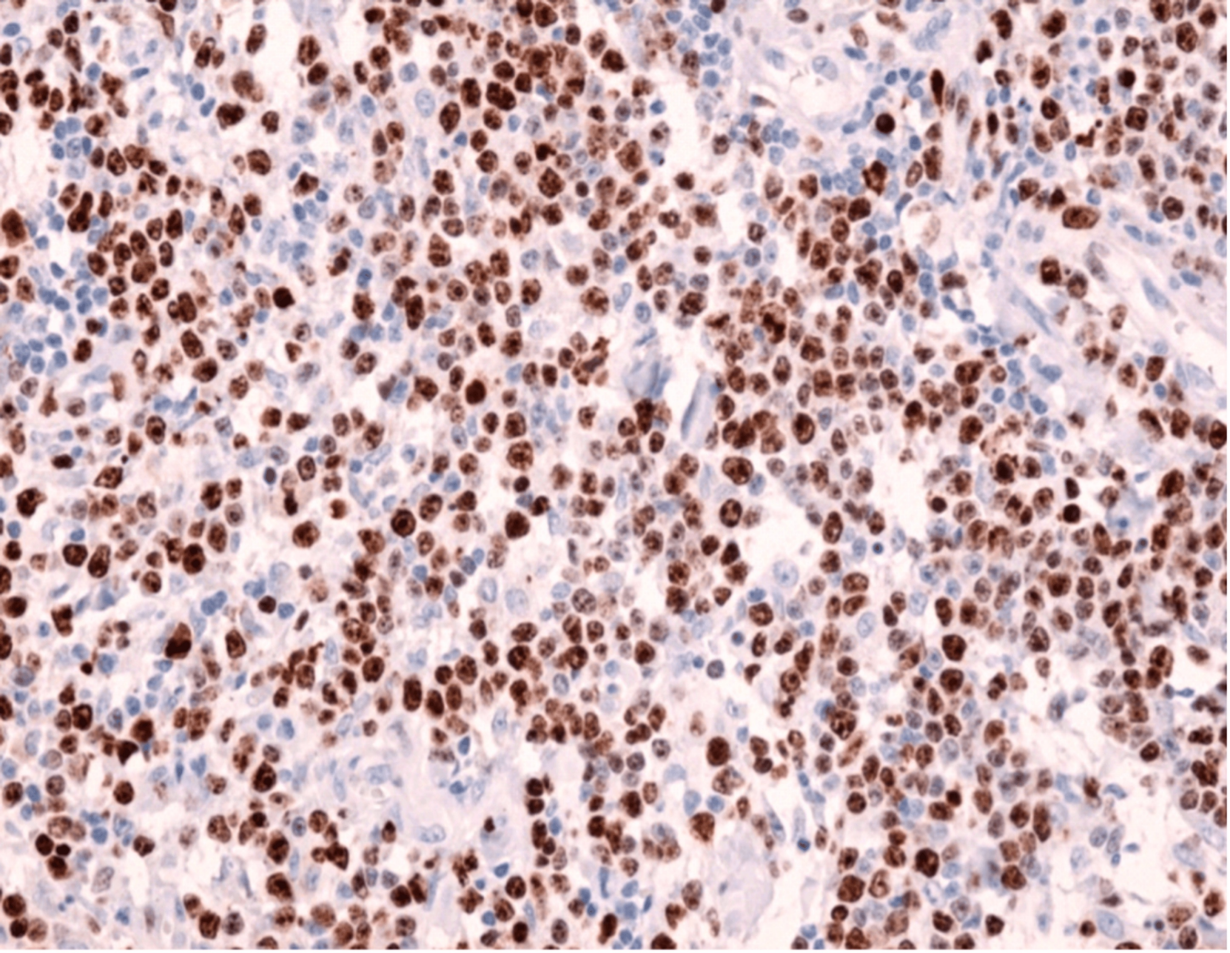
Immunohistochemistry displays positive staining for Ki-67 in more than 70% of the lymphoid cells in diffuse large B-cell lymphoma (magnification x200) Image reproduced with permission. Reprinted from [39]

IHC plays a crucial role in the diagnosis of lymphoma since the lymphocyte type cannot be differentiated on hematoxylin and eosin (H&E) slides. The recommended essential panels for DLBCL include CD3, CD20, Ki-67, CD10, BCL-6, MUM1, BCL-2, C-MYC, and EBV in situ hybridization (ISH) [36]. The Hans algorithm uses CD10, BCL-6, and IRF4/MUM1 markers to differentiate between the germinal center B-cell like (GCB) and the non-GCB subtype [37]. The GCB subtype is CCD10+ or BCL-6+, CD10-, and IRF4/MUM1-, while the non-GCB subtype is CD10- or IRF4/MUM1+ [38]. The Ki-67 index is higher in DLBCL compared to indolent lymphoma due to how it correlates with the degree of aggressiveness of the lymphoma (Figure 4) [6,39].

Management

The current first-line therapy for newly diagnosed DLBCL is multiagent chemoimmunotherapy such as rituximab, cyclophosphamide, doxorubicin, vincristine, and prednisone (R-CHOP). This regimen is curative in approximately 50%-60% of patients, while 30%-40% of patients develop relapse within the first two years of diagnosis. Until recently, salvage chemotherapy followed by stem cell transplantation has been a treatment for patients who develop relapsed disease [40,41]. 

Ki-67

Ki-67 Characteristics

It has been appreciated since the 1920s that cell cycle progression is linked to cell growth. The cell cycle consists of the phases gap (G1), synthesis (S), G2, and mitosis (M). The G1 phase is the most important phase for the regulation of proliferation and differentiation. In the G1 phase, the growth of the cell depends on the presence of external growth factors. Without the growth factors, the cell exits the cell cycle and enters a resting state termed G0. They can remain for days, weeks, or years before resuming proliferation. After the G1 phase, the proliferating cell enters the S phase, during which the DNA is replicated. After the genome is completely replicated, the cell enters the G2 phase. In the G2 phase, the cell prepares for the upcoming mitosis through protein synthesis and the doubling of the centrosome. The M phase is a short phase, lasting about one hour for a cell cycle time of 24 hours [42,43].

Ki-67 was first identified in 1983 as an antigen in Hodgkin lymphoma cell nuclei and is encoded by the gene MK167 [44]. The name of this protein, "Ki,” is based on its city of origin, Kiel, and "67,” referring to its location within the 96-well plate [45]. Ki-67 antigen is a cell proliferation-related non-histone nuclear protein [46]. The protein isoforms encoded by this gene have molecular weights of 345 and 395 kilodaltons (kDa). The Ki-67 protein has a half-life of only 1-1.5 hours [47]. The Ki-67 protein is present in the cell cycle’s active phases (G1, S, G2, and M), but does not exist during the resting state (G0) [48]. Ki-67 levels are low in the G1 and S phases and peak early in mitosis. A sharp decrease in Ki-67 levels occurs in the later phases of mitosis [47]. These characteristics of Ki-67 make it clinically important as a proliferation marker for multiple types of cancer [44]. 

The localization of Ki-67 undergoes variations throughout the cell cycle, and its levels are regulated by transcription and protein degradation that are dependent on the cell cycle. During the G1 and S phases, Ki-67 is found in certain areas within the nucleus, and as the nucleoli are reformed, it becomes concentrated at the periphery. During metaphase, Ki-67 reaches its peak intensity and covers the surface of the chromosomes [49].

Ki-67 as a Diagnostic and Prognostic Tools

Ki-67 is the most widely targeted antigen in pathology among the many proliferation indicators [45]. Since it only reacts with the proliferating cells and has no tissue specificity, it has acted as an accurate marker to determine the proliferative status of tumor cells [50]. The original Ki-67 only worked on frozen tissues and did not work on formalin-fixed or paraffin-embedded tissue. This limits its use in routine practice. Fortunately, some years later, the monoclonal antibody MIB-1 was generated, and the use of Ki-67 IHC became easy [45]. It was first reported in 1992, and it became the most frequently used antibody [51]. MIB-1 recognizes the Ki-67 nuclear antigen and has been proven to be the best proliferation marker in formalin-fixed and paraffin-embedded tissue sections for routine use [52]. The percentage of Ki-67-positive cells is low in benign lesions and high in malignant tumors. This makes Ki-67 an excellent marker to recognize rapidly proliferating cells that would indicate malignancy [46]. Ki-67 has also been used as a prognostic marker in several types of cancer, including thyroid, breast, lung, colorectal, cervical, prostate, and neuroendocrine tumors and NHL. Indeed, a meta-analysis study conducted by Pan et al. found that high Ki-67 expression was a valuable prognostic indicator for NHL and its various subtypes, but not for Hodgkin lymphoma. These studies show that high Ki-67 expression correlates with poorer survival rates [53-60]. A higher quantity of actively dividing cancer cells is typically linked to a more severe clinical course [5].

Ki-67 as a Marker to Differentiate BL and DLBCL

Ki-67 synthesis in all active cell cycles makes it a proliferative marker for a variety of malignancies, including lymphoma [61]. Furthermore, Ki-67 holds the highest level of sensitivity and specificity among the major B lymphoma tumor markers, including CD38 and CD71. Its expression can be evaluated by the proliferation index (PI). The PI is calculated by dividing the number of cells staining positive for Ki-67 by the total number of cells present in the sample [62]. WHO divided the lymphomas into aggressive B-cell lymphoma, indolent B-cell lymphoma, and transformed groups. According to the study by Mao et al., Ki-67 expression was most pronounced in aggressive lymphomas, followed by transformed lymphomas, and the lowest in indolent lymphomas [37]. Thus, the Ki-67 positive level is consistent with the lymphoma grade [6]. In a study by Ali et al., the cutoff value for distinguishing indolent from aggressive lymphomas was 45% [63]. Broyde et al. evaluated the differences in mean Ki-67 index from 26.6% for indolent lymphomas, 67.2% for aggressive lymphomas, and 97.6% for very aggressive lymphomas. They used a documented Ki-67 index of 45% to distinguish between indolent and aggressive lymphomas [64]. A study found that a Ki-67 index greater than 45% is defined as aggressive lymphoma, with a sensitivity of 85% and a specificity of 88.8%. Rebiere et al. conducted a study of Ki-67 expression in DLBCL, and the results showed that the median Ki-67 positivity was 80% [65]. DLBCLs and BLs had a higher Ki-67 index compared with other indolent small cell lymphomas, whereas BL had the highest Ki-67 percentage [6]. The distinction between DLBCL and BL is important because it suggests different treatments [63]. IHC has helped not only to establish a diagnosis but also to differentiate a diagnosis, one of which is Ki-67 [66]. The Ki-67 PI for DLBCL is high, usually much more than 40%, and in some cases may be greater than 90%. A study by Zeggai et al. showed all of their DLBCL cases had a Ki-67 index of >80% [61]. Consistent with the other studies, El-Sabah et al. assessed that the mean of Ki-67 PI was 62.40% [67]. BL has a very high proliferation rate, with nearly 100% Ki-67 positive cells [20]. All of the BL cases assessed by Mudassar et al. had >95% Ki-67 [66]. A study by Meena et al. shows the mean Ki-67 PI from DLBCL was 58.02%, while BL was 94.3%. BL had the highest mean Ki-67 index among the other NHLs [68]. Chong et al. used a cutoff of 97.9% Ki-67-positive cells with 98.1% sensitivity and 100.0% specificity to differentiate BLs and DLBCLs [5].

The diagnosis of BL and DLBCL is more complex when implemented than in principle; despite that, various methods may assist in establishing the diagnosis, including histomorphology, immunophenotyping, and genetic testing [1,69]. Immunophenotyping assays frequently used in diagnosing BL and DLBCL include pan-B-cell markers (CD19, CD20, CD22, CD79a, and PAX5), BCL-2, BCL-6, MUM-1, CD10, ki-67, and c-MYC [6,22,69]. Even so, the application of immunophenotyping in diagnostic evaluation may still establish uncertainty; thus, additional tests may be required [69]. The MYC gene translocation, easily identifiable using fluorescence in situ hybridization (FISH), is a defining genetic characteristic of BL. It is also present in 15%-30% of DLBCL patients, who are often associated with BCL-2 or BCL-6 translocations, leading to "double-hit" lymphoma [22,70]. The ISH for EBV-encoded RNA (EBER) can identify the correlation between BL and DLBCL with Epstein-Barr virus (EBV) infection. DLBCL expresses LMP1, LMP2, and EBNA2, whereas BL does not express LMP1 or EBNA2 but does express EBNA1 [71]. 

## Conclusions

The distinction between BL and DLBCL holds significant importance since it directly impacts the therapeutic approach for the patient. However, differentiating between BL and DLBCL in practical application poses greater challenges than in theory. IHC, such as Ki-67, plays an important role in pathology. Ki-67 IHC serves as a diagnostic and prognostic tool. It has the potential to serve as a valuable tool for distinguishing between BL and DLBCL. According to the review conducted, Ki-67 can be a beneficial tool when used alongside various other tests.
